# Improvement of the Laser-Welded Lap Joint of Dissimilar Mg Alloy and Cu by Incorporation of a Zn Interlayer

**DOI:** 10.3390/ma13092053

**Published:** 2020-04-28

**Authors:** Jun Dai, Banglong Yu, Qingdong Ruan, Paul K. Chu

**Affiliations:** 1 Changshu Institute of Technology, School of Automotive Engineering, Changshu 215500, China; yubanglong@163.com; 2 Department of Physics, Department of Materials Science and Engineering, and Department of Biomedical Engineering, City University of Hong Kong, Tat Chee Avenue, Kowloon, Hong Kong; qingruan@cityu.edu.hk (Q.R.); paul.chu@cityu.edu.hk (P.K.C.); 3 School of Mechanical and Electrical Engineering, Soochow University, Suzhou 215021, China

**Keywords:** laser welding, dissimilar metals, magnesium alloy, first-principles calculation, interlayer

## Abstract

During pulsed laser welding of AZ 31B magnesium (Mg) alloy and T2 pure copper (Cu), Cu_2_Mg and Mg_2_Cu are generated, but the bonding ability of the two compounds is usually weak, resulting in low strength. In order to improve the joint of two dissimilar metals, a zinc interlayer was inserted between the Mg alloy and Cu, and the effects of the thickness of the Zn interlayer on the microstructure and properties of the joint were studied. The fused zone consisted of Cu_2_Mg and MgZn, and, according to first-principles calculation, in the same energy range, the area enclosed by the density of the state curve of MgZn was larger than that of Cu_2_Mg. Hence, the bonding ability of MgZn was better than that of Cu_2_Mg, and MgZn improved the strength of the welded joint. The most advantageous thickness of the Zn interlayer was 0.1 mm, and the shear strength was 48.15 MPa that was 161% higher than that of the directly welded Mg/Cu joint.

## 1. Introduction

In many engineering applications, multiple metals are required [1,2]. For example, Mg and Cu alloys are widely used in the aerospace, automobile, and manufacturing industry. Mg and Mg alloys have a low density, high specific strength, good heat dissipation, lightweight, and so on [3,4,5], whereas Cu and Cu alloys have good electrical conductivity, thermal conductivity, and heat dissipation [6,7,8]. The hybrid structure of Mg/Cu dissimilar materials not only satisfies the requirements of electric conductivity, thermal conductivity, and corrosion resistance but also meets the demand for lightweight. Since Mg/Cu hybrid structure have many researches and applications [9,10,11], it is crucial to study the Mg/Cu welded joints [12].

AZ31B Mg alloy and T2 Cu are typically welded by solid-phase diffusion welding [13]. By adjusting the conditions, the thickness of the interfacial layer between Mg and Cu can be optimized, and the shear strength can reach 66 MPa. Du et al. [14] performed transition liquid phase diffusion welding of AZ31B Mg alloy and T2 Cu, and a diffusion zone with a thickness of 450 μm was formed. However, the low plastic fracture was observed at the interfacial diffusion layer. TIG welding of AZ31B Mg alloy and Cu was carried out with a Fe middle layer by Liu et al. [15,16], but the joint strength was not high. In fact, the heat input from the welding arc to the Cu, Fe, and Mg alloys must be controlled strictly, thus imposing limitations in some applications. Tan et al. [17,18] conducted laser filled wire brazing on T2 pure Cu and AZ31B Mg alloy and studied the structure, distribution, and joint characteristics near the Mg/Cu interface, and brittle fracture occurred in the intermetallic compound. AZ31B Mg and T2 pure Cu have been welded by ultrasonic transient liquid phase welding [19]. MgZn, MgCu, CuZn, and CuMgZn have been produced in the joint, and the ultrasonic process has refined the microstructure. The fracture mode of the welded joint has been inferred to be a brittle one between CuMgZn and Cu_5_Zn_8_.

In spite of these aforementioned and other studies, there are still problems with welding of dissimilar metal systems, such as Mg/Cu, and the use of laser welding of Mg/Cu has not been investigated extensively. Herein, a Zn foil was used as an interlayer for laser welding of Mg/Cu, and the effects of the Zn interlayer thickness were evaluated systematically. In addition to experiments, the first-principles calculation was carried out to analyze the electronic structure and properties of the joint, as well as strengthening mechanism.

## 2. Experimental Details

The base metal was the AZ31B Mg alloy with a thickness of 1 mm and lateral dimensions of 100 mm × 50 mm, and the T2 pure Cu plate had a thickness of 0.5 mm. The composition of the two metals is shown in Table 1 and Table 2. Before welding, the plate was ground to get rid of surface oxide, scrubbed with alcohol, and dried. Zn foils with thicknesses of 0.05 mm, 0.1 mm, 0.2 mm, and 0.5 mm were used, and the lateral size was 10 mm × 50 mm.

**Figure 1:**
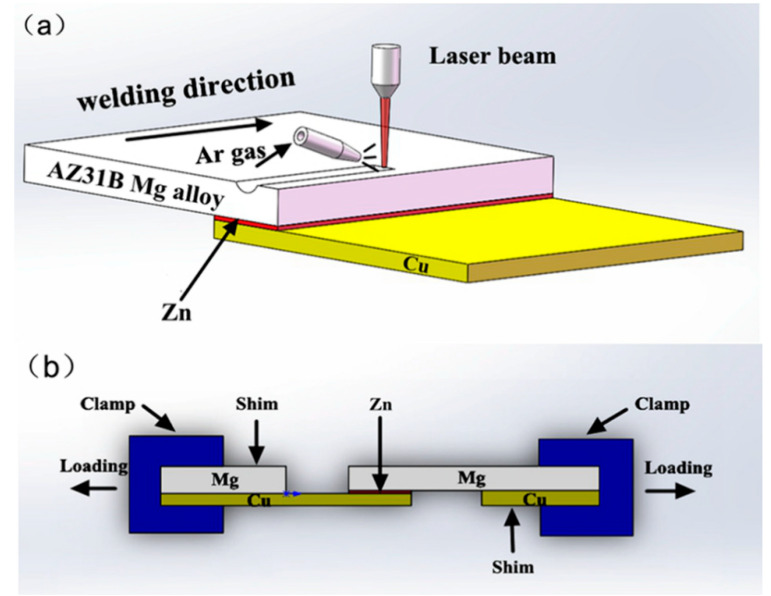
Schematic of the welding experiment and shear test: (**a**) Laser welding and (**b**) Shear test.

The welding equipment consisted of a pulsed Nd: YAG laser (WF-300, Shenzhen, China) with an instantaneous laser power of 2.5 kW, a welding rate of 1.4 mm/s, single-point energy of 19.31 J, and an Ar gas flow rate of 10 L/min. The laser beam diameter was 0.1 mm. The Zn foil was inserted between the Mg plate and Cu plate, as shown in Figure 1. Figure 1a shows a schematic of the welding experiment. The same welding parameters were adopted for the samples with different Zn layer thicknesses, and Figure 1b illustrates the shear test of the welded joint.

After welding, the samples were embedded in epoxy resin, ground, and polished. The microstructure was observed by optical microscopy (OM, OLYMPUS GX51, Tokyo, Japan), scanning electron microscopy (SEM, ZEISS SIGMA500, Dresden, Germany), and transmission electron microscopy (TEM, JEM-2100, Tokyo, Japan). The elemental distributions at the interface and fracture surface were determined by energy-dispersive X-ray spectrometry (EDX). Vickers hardness measurement (HV-10MP, Shanghai, China) was performed across the laser-welded joints with 0.1 mm Zn interlayer under a test load of 100 g and a dwell time of 10 s. The shear strength was determined on the universal testing machine for materials (SUNS UTM 5305, Shenzhen, China) at a rate of 0.05 mm/min in triplicate to obtain the average. The load was increased during the test and then decreased suddenly.

First-principles calculation was conducted based on the density-functional theory and supersoft pseudopotential method [20,21]. The crystal structure of Cu_2_Mg and MgZn was constructed, and the bonding ability was calculated. The CASTEP module in Materials Studio software was implemented to perform generalized gradient approximation (GGA) on the crystal structure, and the equation was self-consistently solved by Kohn–Sham. Before the calculation, the unit cell constant of the compounds and each atomic lattice were fully relaxed until the unit cell energy converged to a fixed value. The results were not related to the spin effects.

## 3. Results and Discussion

**Figure 2:**
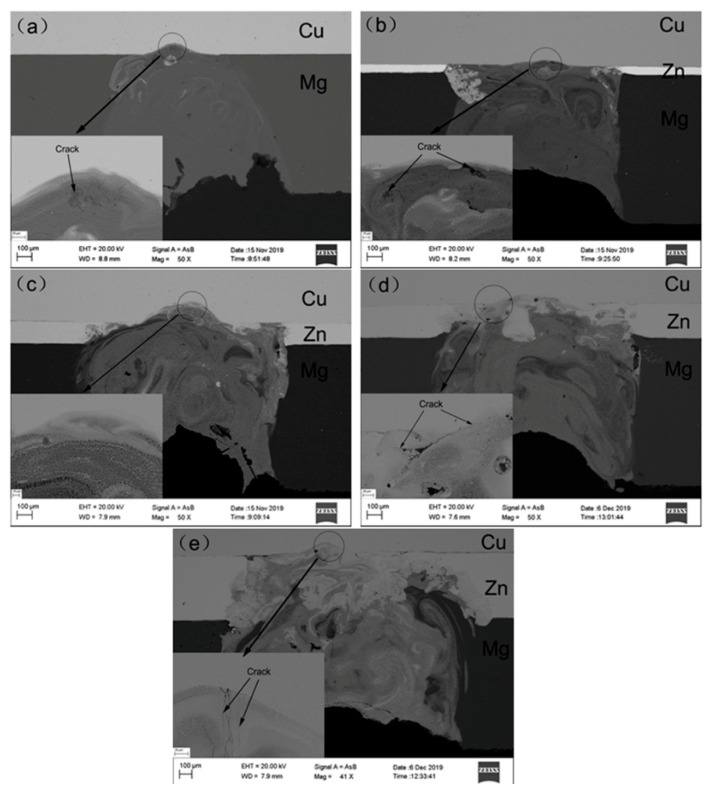
Welding joints with Zn interlayers of different thicknesses: (**a**) Direct welding; (**b**) 0.05 mm; (**c**) 0.1 mm; (**d**) 0.2 mm; (**e**) 0.5 mm.

The welded joints with Zn interlayers of different thicknesses are shown in Figure 2. The magnesium alloy evaporated obviously in the welding process because of the lower boiling point of magnesium (1107 °C), and there was no filler material during the welding, which resulted in the serious concave of the welding joint. Figure 2a,b reveals cracks on the top of the joint, and the cracks in Figure 2d,e were large and long. Figure 2c shows the joint with a 0.1 mm thick Zn interlayer and does not show any cracks and other defects. The elements in the joint were evenly distributed in the joint, and diffusion was relatively uniform.

**Figure 3:**
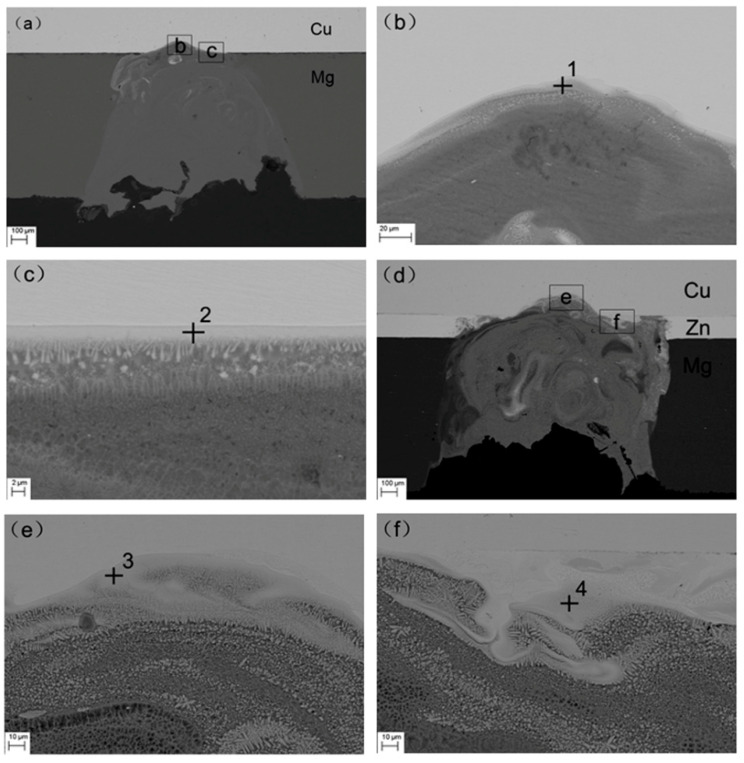
Areas in the welded joint analyzed by EDX: (**a**) Directly welded joint; (**b**) Point 1; (**c**) Point 2; (**d**) Welded joint with a 0.1 mm thick Zn interlayer; (**e**) Point 3; (**f**) Point 4.

Figure 3 shows the areas from which EDX spectra were acquired. Figure 3a–c shows the Mg/Cu joints without an interlayer. Figure 3b is the high-magnification image of area b in Figure 3a, and a small number of cracks could be found. Figure 3c exhibits the high-magnification image of area c in Figure 3a, revealing a smooth intermetallic compound (IMC) layer. Figure 3d–f shows the welded joint with the 0.1 mm thick Zn interlayer. Figure 3e shows the magnified image of area e in Figure 3d, and the joint was fused uniformly without cracks. Figure 3f is the high magnification image of the area f shown in Figure 3d. It could be seen that there was not an obvious smooth IMC layer, and the elements were fully fused.

The EDX results of the four points shown in Figure 3 are presented in Table 3. According to EDX, Mg_2_Cu was formed at point 1 and Cu_2_Mg at point 2. The intermetallic compounds—Mg_2_Cu and Cu_2_Mg—were produced during direct welding of Mg/Cu [22]. Mg_2_Cu was usually generated at the top of the joint, and Cu_2_Mg at the edge. Cu_2_Mg + MgZn was detected from point 3, and MgZn was observed from point 4. Zn prevented the formation of some MgCu intermetallic compounds. According to the phase diagram of the Mg–Zn binary alloy [23], the eutectic reaction between Mg and Zn occurred at 340 °C, and the main intermetallic compound was MgZn.

**Figure 4:**
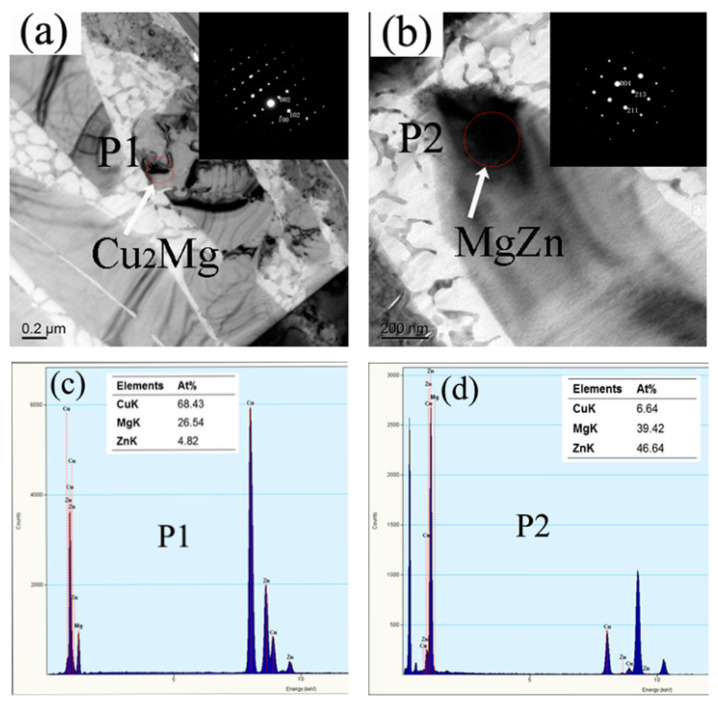
TEM investigation of the joint: (**a**) Bright-field TEM micrograph and SAED patterns of P1; (**b**) Bright-field TEM micrograph and SAED pattern of P2; (**c**) EDX spectrum of P1; (**d**) EDX spectrum of P2.

To identify the phases formed in the fused zone in the joint with the Zn interlayer, TEM was performed. Figure 4 shows the bright-field images together with the EDX and SAED patterns. Figure 4a,b shows the newly formed phases (P1 and P2) in the fused zone. According to Figure 4c,d, the phase at P1 shown in Figure 4a contained 68.43 at% Cu, 26.54 at% Mg, and 4.82 at% Zn and that at P2 shown in Figure 4b consisted of 6.64 at% Cu, 39.42 at% Mg, and 46.64 at% Zn. The phases at P1 and P2 corresponded to Cu_2_Mg and MgZn, respectively, and Zn could react with Mg and inhibit the formation of Mg/Cu compounds.

**Figure 5:**
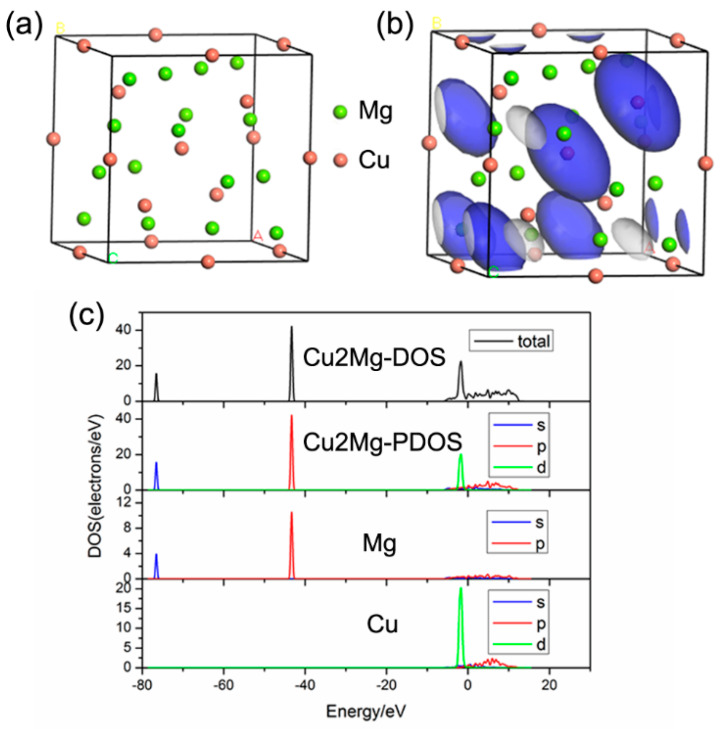
First-principles calculation results of Cu_2_Mg: (**a**) Unit cell model; (**b**) Electron density map; (**c**) State density maps.

**Figure 6:**
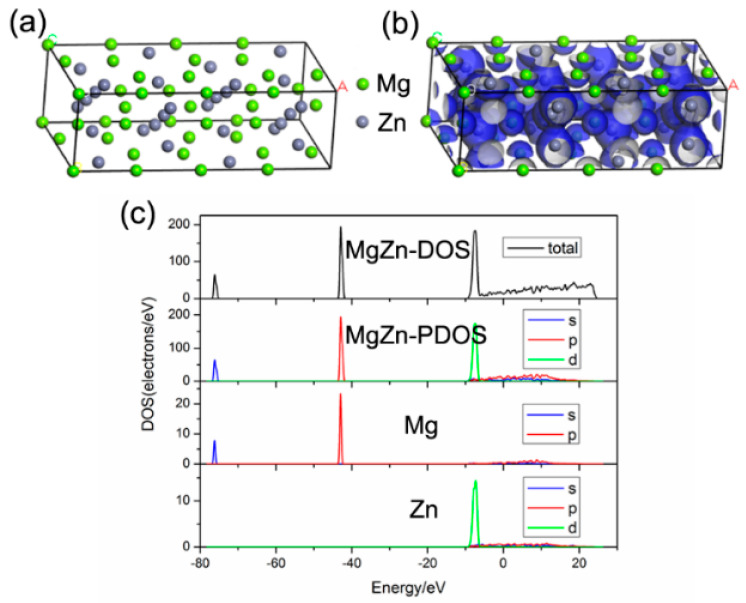
First-principles calculation results of MgZn: (**a**) Unit cell model; (**b**) Electron density map; (**c**) State density maps.

The first-principles calculation was conducted based on the DFT and super soft pseudopotential method. The crystal structures of Cu_2_Mg and MgZn were constructed, and the electronic structures were calculated. The density of state (DOS) map was the visualization result of band. Because the electronic energy levels (orbits) were very dense, a standard continuous energy band was formed. DOS was to summarize the distribution of energy levels. A molecule is formed by two atoms. Two atomic orbitals form two molecular orbitals according to a certain relationship, and two molecular orbitals form two energy bands. For bonding orbitals, it is mainly the atomic orbitals of atoms with higher electronegativity, and the atomic orbitals of other atoms are doped, so the energy band can’t show the contribution of a specific orbit, but the density of state diagram can get the contribution of a specific orbit. The density of state (DOS) can be divided into the local density of state (LDOS) and the partial density of state (PDOS). The LDOS refers to the contribution of the electronic states of each atom to the total density of the state diagram. The PDOS refers to the further resolution of these contributions according to the angular momentum (s, p, d, f). It is determined that the main peak of the density of the state is specifically from the contribution of electronic states, such as s, p, d, or f. According to the PDOS, it can not only analyze the bonding but also analyze where the electron is. The results of Cu_2_Mg are presented in Figure 5. The atomic distribution of Cu_2_Mg was relatively scattered, and the DOS of the p orbital showed a large peak. The energy of the electrons contributing to bonding was mainly concentrated in the range between −50 eV and −40 eV and mainly came from the contributions of valence electrons of Mg (P), Mg (d), and Cu (d). However, the contribution of valence electrons of Mg (s) and Cu (P) was small. Figure 6 shows the results of the MgZn. As shown in Figure 6c, the density of the state diagram of MgZn was similar to that of Cu_2_Mg, but it showed two higher peaks. The vertical coordinate in the density of the state diagram was larger. According to the total density of the state diagram of the two compounds, the area under the density of the state curve was calculated by origin integral, and the number of bonding electrons of the two compounds could be obtained. In the range of −80 ev to 20 ev, the enclosure area of Cu_2_Mg was 111.942, while that of MgZn was 1177.618. Cu_2_Mg crystal structure had 24 atoms, while the MgZn crystal structure had 47 atoms. The bonding electron number of Cu_2_Mg was 4.664, and that of MgZn was 25.055. It was found that the number of bonding electrons of MgZn was much larger than that of Cu_2_Mg, which indicated that the interaction between valence electrons of MgZn was stronger, and the structure was more stable. In the same energy range, the area enclosed by the density of the state curve of MgZn was larger than that of Cu_2_Mg and, hence, the bonding ability of MgZn was better than that of Cu_2_Mg [24,25]. When a Zn interlayer was inserted, the generated MgZn inhibited the formation of Mg/Cu compounds. Consequently, the bonding ability of MgZn was stronger than that of Cu_2_Mg, and the strength of the welded joint was improved.

**Figure 7:**
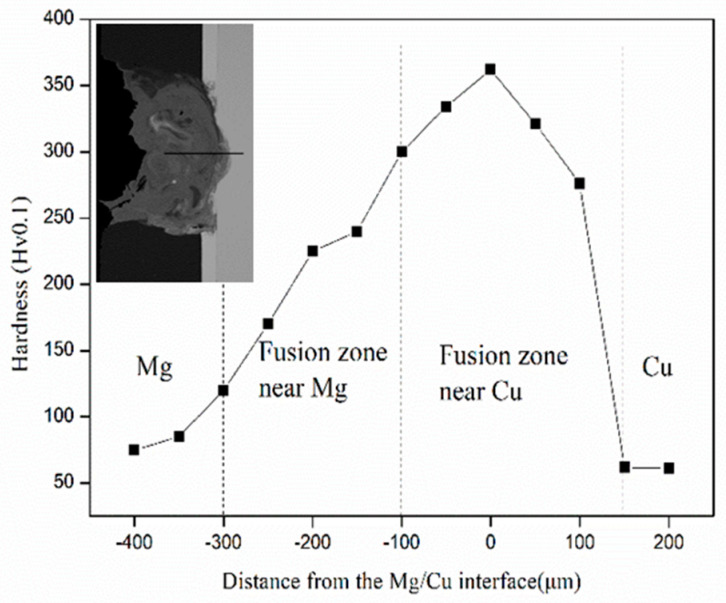
Vickers hardness of the welding joint with 0.1 mm Zn interlayer.

Figure 7 presents the hardness distribution profiles of the Mg/Cu joints. The highest Vickers hardness of the joint was about 360 HV. The Zn interlayer melted in the joints. As could be seen, the hardness of Mg and Cu base metal was 75 HV and 60 HV, respectively. High hardness appeared in the fusion zone near Cu, which was composed of series intermetallics compounds, consistent with Figure 3e. The presence of intermetallic compounds was the main reason for this higher hardness distribution. In the fusion zone near Mg alloy, the average hardness increased to 150–250 HV due to the strengthening effect of the increased Cu content and newly formed Mg–Cu–Zn mixture compositions.

**Figure 8:**
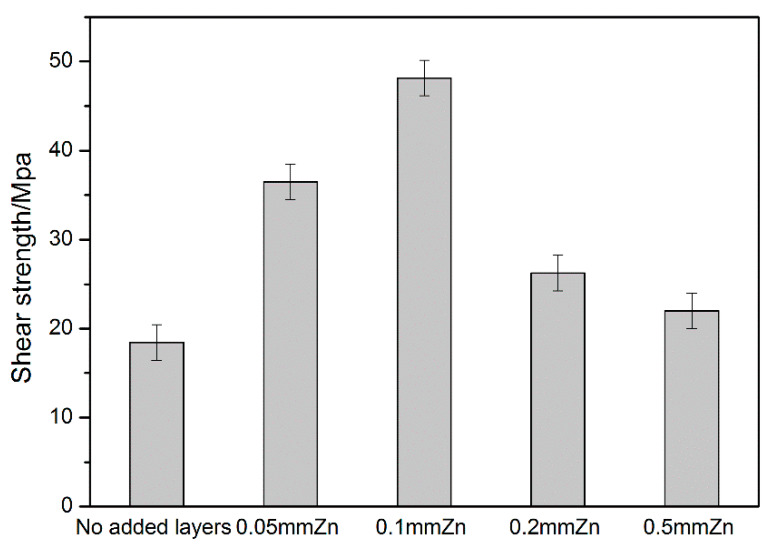
Shear strength of the different welded joints.

**Figure 9:**
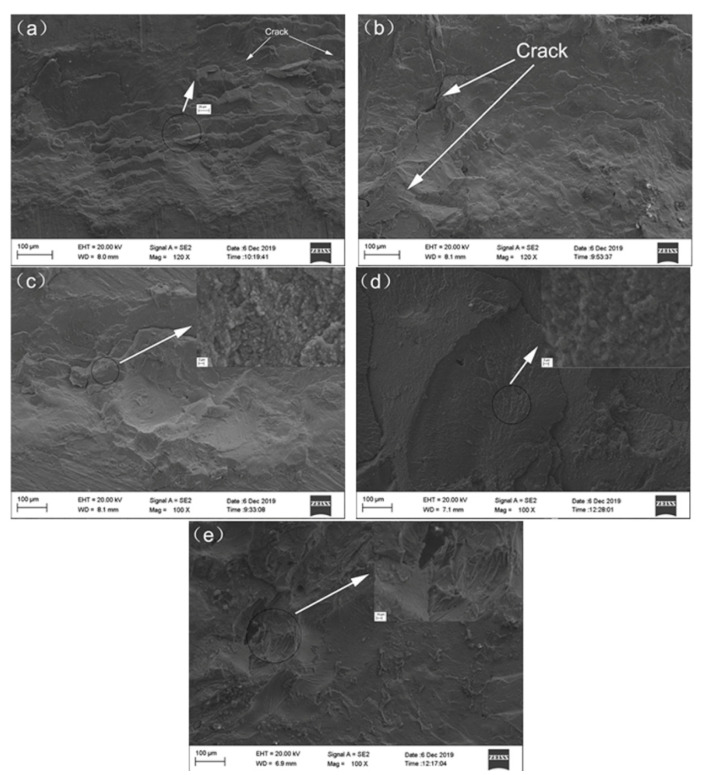
Fractures of the different welded joints: (**a**) Directly welded joint; (**b**) Joint with a 0.05 mm thick Zn interlayer; (**c**) Joint with a 0.1 mm thick Zn interlayer; (**d**) Joint with a 0.2 mm thick interlayer; (**e**) Joint with a 0.5 mm thick Zn interlayer.

The shear strength of the joints is shown in Figure 8. The shear strength of the joint with the 0.1 mm thick Zn interlayer was 48.15 MPa, which was 161% larger than that of the directly welded Mg/Cu joint. When the thickness of the Zn interlayer was increased, the shear strength increased initially but declined afterward. The shear strength of the joint with the 0.5 mm Zn interlayer was 21.98 MPa. The fractures of the different joints are displayed in Figure 9, and Figure 9a,b reveals cracks that reduce the shear strength. As shown in Figure 9c,d, there were torn edges in both joints, and that in Figure 9c was more obvious, indicating that the shear strength of the welded joint with the 0.1 mm Zn interlayer was the highest.

## 4. Conclusions

Strong joints were obtained by pulsed laser welding of Mg/Cu by inserting a 0.1 mm thick Zn interlayer. The optimal welding parameters were instantaneous laser power of 2.5 kW, a welding rate of 1.4 mm/s, single-point energy of 19.31 J, and an Ar gas flow rate of 10 L/min. Intermetallic compounds—Mg_2_Cu and Cu_2_Mg—were produced during direct welding of Mg/Cu. Mg_2_Cu was usually generated at the top of the joint, whereas Cu_2_Mg was generated at the edge. Cu_2_Mg and MgZn were formed in the fused zone in the joint with Zn as the interlayer, and Zn prevented the formation of some Mg/Cu intermetallic compounds. The bonding ability of MgZn was greater than that of Cu_2_Mg, according to first-principles calculation. Experimentally, the shear strength of the welded joint with the 0.1 mm thick Zn interlayer was 48.15 MPa, which was 161% higher than that of the directly welded Mg/Cu joint.

## Figures and Tables

**Table 1:** Chemical composition of AZ31B Mg alloy (wt%).

Alloy	Al	Zn	Mn	Si	Fe	Cu	Ca	Mg
**AZ31B Mg**	3.19	0.81	0.334	0.02	0.005	0.05	0.04	Bal.

**Table 2:** Chemical composition of T2 Cu (wt%).

Alloy	Fe	O	Ni	S	Pb	Bi	Ti	Cu
**T2 Cu**	0.005	0.06	0.006	0.001	0.001	0.001	0.006	Bal.

**Table 3:** EDX results of the reaction layers described in Figure 4 (at%).

Elements	Cu	Mg	Zn
Spectrum 1	32.39	67.61	--
Spectrum 2	65.95	34.05	--
Spectrum 3	54.82	33.35	11.83
Spectrum 4	12.05	41.32	46.63
